# A multidimensional education program at substance dependence treatment centers improves patient knowledge and hepatitis C care

**DOI:** 10.1186/s12879-016-1883-6

**Published:** 2016-10-12

**Authors:** Rui Tato Marinho, António Costa, Teodomiro Pires, Helena Raposo, Carlos Vasconcelos, Cristina Polónia, Joaquim Borges, Mariana Soares, Graça Vilar, Ana Maria Nogueira, on behalf of the LIGUE-C Investigators, Rosa Mateus, Alcides Rodrigues, João Domingues, Paula Coelho, Paula Santos, Manuel Seoane, Mariana Coelho, José Correia, Luísa Teixeira, Miguel Vasconcelos, Hélder Costa, Luís Nascimento, Marta Andrade, Rosário Vasconcelos, Carla Proença, Marta Cadete, Rita Carvalho, Carla Gonçalves, Denise Rocha, Alexandra Camilo, Elsa Costa, Douteiro Sá, Cristina Recalde, Isabel Vila Nova, Lisete Feijó, Sara Passos, Silvia Ribeiro, Sandra Peixoto, Armando Melo, Diana Pacheco, Ana Teles, Anabela Almeida, Patrícia Abreu, Sónia Antunes, Sónia Paiva, Ana Gabriela Silva, José Fernandes, Célia Carvalho, Nídia Rosa, Stela Camarneiro, Nélia Capêto, Fernanda Rodrigues, Ana Luísa Góis, Carmen Fernandes, Lina Correia, Paula Carrinho, Paula Boal, Margarida Gil, Cristina Fonseca, Ana Nunes, Luís Tavares, Ana Paula Tavares, Ana Horta, Fátima Augusto, Cláudia Cardoso, Abílio Gonçalves, Pilar Fernandez, Rita Herculano

**Affiliations:** 1 Department of Gastroenterology and Hepatology, Faculty of Medicine, University of Lisbon, Hospital Santa Maria - Centro Hospitalar Lisboa Norte, Av. Prof. Egas Moniz, Lisbon, 1649-035 Portugal; 2 UD Centro das Taipas, Parque de Saúde de Lisboa – Av. Brasil n.° 53, Pavilhão 2, 1° andar, 1749-002 Lisbon, Portugal; 3 ETET de Almada, Rua das Terras dos Cortes Reais n°1, 2805-025 Almada, Portugal; 4 ETET do Barreiro, Rua Almirante Reis n°50, 2830-326 Barreiro, Portugal; 5 ETET de Gondomar, Rua Caminho de Pevidal, R/C – S/N, 4420-264 Gondomar, Portugal; 6 ETET de Setúbal, Praça da República, 2900-587 Setúbal, Portugal; 7 ETET da Figueira da Foz, Rua Doutor Calado 2, Figueira da Foz, 3080-153 Figueira da Foz, Portugal; 8 ETET Eixo Oeiras Cascais, Rua Professor Orlando Ribeiro, n.°3A, B, n.° 5A, B e n.° 7, 2740-222 Porto Salvo, Portugal; 9 SICAD- General-Directorate for Intervention on Addictive Behaviours and Dependencies, Avenida da República, n° 61, 3° piso, 1050-189 Lisbon, Portugal; 10 MSD Portugal, a subsidiary of Merck & Co., Inc., Quinta da Fonte, Edifício Vasco da Gama, 19, 2770-192 Paço de Arcos, Portugal

**Keywords:** Hepatitis C, Health education program, Treatment access, Substance abuse, People who inject drugs, Chronic hepatitis C treatment

## Abstract

**Background:**

HCV treatment among people who inject drugs (PWID) is low. Education programs may be suitable strategies to improve patients’ knowledge about their condition and to overcome barriers to access treatment.

**Methods:**

The Health Educational Program (HEP) consisted of patient workshops and educational videos and leaflets, and healthcare professionals’ workshops. HEP was implemented at seven substance dependence treatment centers (STDC) in Portugal. The study comprised two cross-sectional evaluations conducted before and after HEP. At both evaluations, adult patients with confirmed HCV diagnosis and registered in the STDC were consecutively included. For patients that completed both evaluations, the overall knowledge score were calculated and compared with McNemar test. Linear regression modelling was used to evaluate factors associated with baseline knowledge. Rates of referral and attendance to referral specialist, treatment proposal, initiation and retention at both evaluations were also compared with McNemar test.

**Results:**

Overall, 504 patients with chronic hepatitis C were included: 78 % male, mean age 42.3 ± 6.6 years, 14 % school education ≤ 4 years, disease duration 11.0 ± 6.0 years and 26 % HIV co-infected. A higher baseline knowledge was independently associated with educational level ≥ 10 years (regression coefficient [B] =15.13, *p* < 0.001), current use of intravenous drugs (B = 7.99, *p* = 0.038), previous referral for treatment (B = 4.26, *p* = 0.008) and previous HCV treatment (B = 5.40, *p* = 0.003). Following HEP, mean knowledge score increased from 69 % to 79 % (*p* < 0.001). The rate of patient referral to a liver specialist increased from 56.2 % to 67.5 % (*p* < 0.001).

**Conclusions:**

An HEP conducted at STDCs improved significantly patient knowledge about hepatitis C, even among patients with a high baseline knowledge. The HEP has also increased the rate of referral to the liver specialist and showed a great potential to support healthcare professionals in managing HCV. Education programs may promote treatment access among PWID, a population that represents the majority of HCV infected patients.

**Electronic supplementary material:**

The online version of this article (doi:10.1186/s12879-016-1883-6) contains supplementary material, which is available to authorized users.

## Background

Hepatitis C virus (HCV) infection is a major public health concern with 130–150 million people infected worldwide [1, 2]. Approximately 50–80 % of acute HCV infections progress to chronic disease. If untreated, 20–40 % of patients with chronic hepatitis C (CHC) will develop cirrhosis within 25 years [3]. Decompensated liver disease and hepatocellular carcinoma occur in 25 % of late stage cirrhotic patients [3]. Extrapolation from blood donors suggests a HCV prevalence of approximately 1.5 % in the Portuguese population [4, 5] and the estimated rate of diagnosis among HCV infected is approximately 30 % [6, 7].

People who inject drugs (PWID) are the largest group of infected persons. Reports from the World Health Organization point out to a prevalence of HCV infection among PWID of 46 % in the European region [4]. In developed countries, despite the disease burden and high transmission risk among PWID, only 20–30 % of these patients are receiving treatment for hepatitis C [8, 9]. In fact, even though HCV treatment regimens have been simplified, special attention remains necessary towards the promotion of patient access to treatment [10–12].

Barriers to access to HCV treatment are multifactorial and related to the healthcare system and to both healthcare professionals and patients [8]. In Portugal, there are no specific guidelines for treating PWID with HCV infection, although national HCV guidelines recommend the treatment of active intravenous drug users due to their increased transmission risk [13–15]. Regarding Portuguese healthcare professionals, it has been pointed out that physicians should update their knowledge concerning hepatitis C [16], as it may contribute to improving patient access to HCV care [17–19].

On the other hand, the optimization of the HCV care may also be influenced by patient knowledge about their condition, as well as their attendance to medical appointments and adherence to HCV treatment, which, in turn, may translate into increased cure rates [20]. Patients’ resistance to HCV treatment is likely related to fear of procedures such as liver biopsies, and misconceptions about treatment side effects. Awareness about disease morbidity and mortality has been low, and treatment is often avoided and perceived as discretionary [21, 22]. Effective educational interventions can potentially reduce HCV transmission and improve outcomes in vulnerable populations [12]. Typically, HCV educational programs are multidisciplinary and may use different approaches [22, 23].

In Portugal, treatment of drug addiction involves outpatient drug treatment, day-care centers, detoxification units and therapeutic communities. Substance dependence treatment centers (SDTCs) provide outpatient drug treatment and are the preferred unit for screening, treatment and follow-up of these patients [21, 24]. All STDCs provide psychosocial and substitution treatment, as well as screening of HIV, HBV and HCV [25]. In 2012, HCV infection rate was 61 % among drug users and 88 % among PWID followed at STDCs [25, 26]. There is a lack of information about the uptake of HCV treatment among PWID in Portugal [9, 27] although, regarding overall HCV patients, a 2013 expert panel has estimated that only 30–40 % ever received treatment [6].

Despite the epidemic rates of infectious diseases among drug users, especially CHC in PWID, and the low uptake of HCV treatment in Portugal, little is known about educational strategies to optimize the HCV care among these populations.

Our research aimed to investigate the impact of a multidimensional Health Educational Program (HEP) implemented at SDTCs in Portugal on patient knowledge about hepatitis C and on HCV clinical care. Furthermore, we investigated the factors most associated with baseline knowledge about CHC.

## Methods

### Study design

This was an interventional study with two cross-sectional evaluations before and after the HEP implementation: Phase 1 (baseline assessment) was conducted during the first STDC appointment from April to September 2012, before the implementation of the HEP. Phase 2 (post-education assessment) was conducted during the first STDC appointment after the implementation of the HEP, from February to December 2013. The study was approved by the Ethics Committee of the *Centro Hospitalar Lisboa Norte - Hospital de Santa Maria*. All patients provided written informed consent at both evaluations.

### Setting

The study was conducted at seven SDTCs in mainland Portugal. Treatment teams at the STDCs are multidisciplinary and include psychologists, physicians, social assistants, nurses, medical auxiliaries and psychosocial technicians [21]. Public SDTCs are accessible to all drug users without incurring any costs. In 2012, there were 80 STDC in Portugal following approximately 29,062 individuals, mainly from Lisbon (29 %) and Oporto (23 %) regions [25]. In this study, the seven STDCs were distributed across the regions of Lisbon (5), Oporto (1) and Coimbra (1).

### Participants

Patients aged 18 years or older with confirmed diagnosis of HCV infection, registered in the STDC were consecutively included in the study during appointments at the STDC at phase I and phase II. Patients had no financial compensation for participating in the study. Absence of records about HCV diagnosis was an exclusion criterion. The participants who completed both cross-sectional evaluations were included in the analysis.

### Health education program

A health education program (HEP) may be defined as an action or a group of actions aiming to achieve certain health-related desired effect such as increased disease awareness, improved treatment knowledge or behavioral change [28]. The 6-month HEP was addressed to patients and healthcare professionals, and included several components (Table 1).

**Table 1:** Description of the different components of the health education program

Educational initiatives for patients and caregivers:
• Educational videos and leaflets were available in SDTCs’ waiting rooms, with information about HCV natural history, treatment options, expected outcomes and management of side effects.
• Supervised patient workshops facilitated by treatment community healthcare professionals aimed to promote open discussions among patients and explain any topics about HCV infection.
Educational activities for healthcare professionals in referral hospitals and in SDTCs:
• Workshops for healthcare professionals at SDTCs, namely psychiatrists, psychologists, nurses and social workers. • Conducted by specialists about HCV and substance dependence. • Presentation and discussion of clinical cases.

Patients attending to STDCs during the HEP were exposed to educational videos and leaflets with information about hepatitis C. Furthermore, patients were invited to participate in programmed workshops moderated by a Psychologist of the investigational team over the 6-month period. Workshops aimed to promote the discussion and experience sharing among patients and between patients and HCPs about hepatitis C. Workshops lasted approximately one to two hours and addressed pre-specified topics: hepatitis C treatment initiation, patient engagement, compliance monitoring, adverse event management, and psychological and clinical management of difficult-to-treat patients. Each STDC could propose one or more workshops during the HEP, which were freely available to the patients. All HEP materials were developed by two liver specialists, based on similar videos and leaflets, and on the literature review [29].

The workshops for STDC healthcare professionals were conducted by liver specialists of reference hospitals of each STDC region. The following topics were covered during these sessions: initiation of treatment, patient's adherence to the medical appointments, assessment and reinforcement of patient adherence to treatment, side effects management, and psychological and clinical follow-up of non-responders/relapsing patients.

### Variables

Socio-demographic data (age, sex, educational level, marital and employment status, history of drug addiction) and HCV data (mode of transmission, duration, HCV genotype and viral load [when available]) were collected from the SDTCs patients’ records into a structured data collection tool, similar in both study phases. In addition, the following variables were collected at baseline and after HEP: past referrals to HCV treatment, previous HCV treatment and number of appointments to the SDTC, to the reference hospital and to the liver specialist in the previous six months. Current HCV treatment and attendance to HEP sessions were also collected, based on information provided by the hospitals to the STDCs, by email, phone contact or patient written letter.

### Assessment of patient knowledge

Patient knowledge about HCV infection was assessed with a 13-item closed-ended questionnaire during the two study phases. The questionnaire was developed by two liver specialists and covered two domains: general knowledge about HCV with nine questions (including disease progression, definition, mode of transmission, symptoms, outcomes and common comorbidities) and knowledge about HCV treatment with four questions (treatment options, treatment response and side effects). Each participant was interviewed by the same researcher during both phases, whenever possible.

For all questions, there were correct and incorrect options. For six questions, patient had to answer “yes” or “no”. The remaining questions were multiple-choice, of which some had more than one correct option. An overall knowledge score was calculated by assigning one point to each correct answer. This score reflected the percentage of points obtained out of the 13 questions. For questions with multiple choices the following criteria were used (as applicable): a) when a patient chose more options than the correct one a score of “1/number of answers” was attributed; b) when a patient chose one or two out of three correct answers a score of 0.33 and 0.67 was attributed, respectively; c) the score was the number of correct answers over 9 (for one question with nine correct answers). For all questions, missing data and “don’t know” answers were considered as not correct (score = 0). Knowledge sub-scores were also calculated for the disease domain (first nine questions) and treatment domain (remaining four questions).

The impact of the HEP was evaluated by comparing patients’ overall knowledge score, disease and treatment sub-scores and the proportion of correct answers to each question, at the two study phases.

### Assessment of clinical care of HCV

The impact of the HEP program in the clinical care of HCV was measured by comparing the rates of patient referral (physician initiated), attendance at referral appointments, treatment proposal, treatment initiation and treatment retention at baseline and after exposure to the program. The definition of each endpoint of clinical care is shown in Table 2.

**Table 2:** HCV infection clinical care endpoints

Endpoints	Definition
Patient referral rate	Nr. of patient referred to liver specialist^a^/Nr. of patients enrolled in the program
Attendance at referrals rate	Nr. of patients with confirmed hospital visit^b^/Nr. of patient referred to liver specialist
Treatment proposal rate	Nr. of treatment proposals^c^/Nr. of referred patients with confirmed hospital visit
Treatment initiation rate	Nr. of treatment initiations^d^/Nr. of treatment proposals
Treatment retention rate	Nr. patients that completed or maintained treatment^e^/Nr. patients that initiated treatment

*Nr* number

^a^Patient referrals = patients with a previous referral appointment scheduled at a liver specialist

^b^Patients with confirmed hospital visit = Patients who referral resulted in a liver specialist appointment

^c^Treatment proposal = treatment eligible patients who were proposed for treatment by the liver specialist

^d^Treatment initiation = at least one medication intake confirmed by liver specialist

^e^Treatment retention = patients that completed or were still on treatment, whichever occurred first

### Statistical analysis

Mean, median and standard deviation were used for continuous variables. Absolute and relative frequencies were used for categorical variables. The Wilcoxon test was used to compare knowledge scores (overall and sub-scores) between time points. The McNemar test was used to analyze the HCV percent changes in the clinical care endpoints (rates of patient referral, attendance at referrals, treatment proposal, treatment initiation and treatment retention) and the proportion of answers for each question between baseline and post-HEP evaluation.

The association of patient knowledge score at baseline with demographic and clinical variables was evaluated through bivariable analysis, using the non-parametric tests of Mann–Whitney or Kruskal-Wallis for categorical variables and the Spearman correlation for continuous variables. A multiple linear regression model was used to evaluate the factors independently associated with baseline knowledge score, choosing those variables with *p*-value <0.20 and with less than 10 % of missing information. The observed results of the multiple linear regression were valid according to the assumptions about residuals and predicted values.

At the time of the study implementation, there were approximately 4500 patients registered in the seven STDCs (overall). Of these, it was estimated that 50 % were HCV infected. In the analysis, we planned to include approximately 500 HCV-infected patients that completed both the baseline and post-HEP assessments at SDTC. This number allowed to estimate a per cent improvement using a 95 % confidence interval (CI) and a margin of error less than 5 %.

All *p* values were two-sided and the level of significance was set at *p* <0.05 with 95 % confidence intervals, when applicable. Statistical analysis was performed using the Statistical Package for the Social Sciences, version 19.0 (SPSS Inc., Chicago, IL, USA).

## Results

### Sample characterization

Overall, 1080 patients were evaluated in the phase I and 754 were evaluated in the phase II. During phase I, 34 patients were excluded: 30 did not complete the questionnaires and four had no confirmed diagnosis of hepatitis C. From the remaining 1,046 patients, 542 (51.8 %) did not complete the phase II questionnaire and 504 patients performed both assessments, being therefore included in this analysis. Patients that did not participate in phase II were younger (median age 40 vs. 42, *p* = 0.008) and more employed (36 % vs. 29 %, *p* = 0.001) than participants. There were no differences regarding sex and marital status.

Included patients had a mean age of 42.3 ± 6.6 years and the majority were male (78 %) (Table 3). Approximately 14 % of patients had four years or less of school education, 55 % were single and 62 % were unemployed. PWID was the most common route of HCV infection (66 %) and 21 % of patients were unaware how they acquired the infection. Most of the patients (84 %) were receiving opioid substitution therapy. Mean time since HCV diagnosis was 11.0 ± 6.0 years and 26 % of patients were HIV co-infected. At baseline, 68 % of patients had a previous referral to HCV treatment and 21 % had received treatment in the past, with no response to interferon and ribavirin. The mean number of appointments to the SDTC and liver specialists in the previous 6 months was 7.6 ± 7.5 and 1.7 ± 2.3, respectively.

**Table 3:** Socio-demographic and clinical characteristics

Age, years (mean ± SD)	42.3 ± 6.6
Gender - Male, % (n)	78.0 (393)
Educational level, % (n)	
4 years or less	13.7 (69)
5–9 years	62.2 (314)
10 years or more	24.1 (121)
Marital status, % (n)	
Single	54.8 (276)
Married	23.8 (120)
Divorced	20.2 (102)
Widow	1.2 (6)
Professional status, % (n)	
Student	2.0 (10)
Unemployed	61.5 (310)
Employed	28.8 (145)
Retired	6.0 (30)
Other	1.8 (9)
Substance abuse (current), % (n)^a^	
Intravenous drug use	3.6 (18)
Non-intravenous drug use	8.1 (41)
Alcohol abuse	9.1 (46)
Opioid substitution treatment	83.5 (421)
Other	9.9 (50)
HCV Transmission Mode, % (n)^a^	
Intravenous drug use	65.7 (331)
Post-transfusion	1.0 (5)
Peri-natal	0.0 (0)
Sexual	10.5 (53)
Other	2.4 (12)
Unknown	20.6 (104)
Time since HCV diagnosis, years (mean ± SD), *n* = 356	11.0 ± 6.0
HCV Genotype, % (n), *n* = 502	
1	2.4 (12)
1a	0.8 (4)
1b	0.4 (2)
2	1.2 (6)
3	1.6 (8)
4	1.0 (5)
Unknown	92.6 (465)
HCV viral load, RNA, x10^6^ IU/mL (mean ± SD), *n* = 27	0.5 ± 1.5
Comorbidities, % (n)^a^	
HIV	25.8 (130)
Hepatitis B	12.5 (63)
Mental disorders	16.1 (81)
Other	7.5 (38)
Past referrals to HCV treatment, % (n)	68.3 (344)
Past HCV treatment, % (n)	21.0 (106)
Number visits to the SDTC in the last six months (mean ± SD)	7.6 ± 7.5
Number visits to a liver specialist in the last six months (mean ± SD)	1.7 ± 2.3

Unless otherwise stated, *n* = 504

*SDTC* Substance Dependence Treatment Center, *SD* standard-deviation

^a^More than one possible answer

### Patient knowledge regarding HCV

Table 4 shows the patient knowledge about HCV and its treatment prior and after the implementation of the HEP.

**Table 4:** Patient knowledge about Hepatitis C and its treatment, prior and after the health educational program

	Baseline	Post-HEP	*p* value
Overall knowledge score, %^a^			<0.001
mean ± SD	68.8 ± 16.6	79.0 ± 12.4	
median (min-max)	71.8 (7.7–98.3)	81.2 (15.4–100)	
Disease knowledge sub-score, %^a^			<0.001
mean ± SD	71.6 ± 16.4	79.9 ± 13.1	
median (min-max)	74.1 (11.1–100)	81.4 (11.1–100)	
*Questions*			
1. Patient shows symptoms immediately after infection with HCV			
No, % (n) [correct]	78.0 (393)	88.9 (448)	<0.001
2. If left untreated, the majority of cases progress to cirrhosis			
Yes, % (n) [correct]	77.2 (389)	86.1 (434)	<0.001
3. Co-infection with HIV may increase disease progression			
Yes, % (n) [correct]	78.8 (397)	86.7 (437)	<0.001
4. Alcohol consumption may increase disease progression			
Yes, % (n) [correct]	96.8 (488)	98.8 (498)	<0.001
5. What is HCV disease, % (n)			
Is a liver viral disease [correct]	91.5 (461)	96.4 (486)	<0.001
Is a disease that affects whole body organs	3.0 (15)	2.2 (11)	
Doesn’t know	5.6 (28)	1.4 (7)	
6. How is HCV transmitted, % (n)^b^			
Sexual transmission only	4.8 (24)	2.6 (13)	0.061
Sexual and blood transmission [correct]	73.9 (370)	84.3 (425)	0.001
Physical contact	4.6 (23)	3.0 (15)	0.134
Sharing of materials related with drug use	15.6 (78)	10.1 (51)	0.004
Doesn’t know	5.2 (26)	1.4 (7)	<0.001
Other	2.8 (14)	1.4 (7)	
7. What are the symptoms of HCV disease, % (n)^c^			
Generalized weakness [correct]	38.7 (195)	41.7 (210)	0.295
Weight loss [correct]	19.2 (97)	23.8 (120)	0.062
Vomits and diarrhea [correct]	10.3 (52)	13.3 (67)	0.142
All the previous [correct]	19.4 (98)	20.6 (104)	0.659
There are no symptoms	14.5 (73)	16.9 (85)	0.261
Doesn’t know	21.2 (107)	14.5 (73)	0.001
8. HCV disease is curable			
Yes, % (n) [correct]	74.2 (373)	88.7 (447)	<0.001
9. Which diseases are most associated with HCV disease?, % (n)^c^			
HIV	20.2 (102)	31.7 (160)	<0.001
Hepatitis B	25.4 (128)	27.6 (139)	0.396
Tuberculosis	8.7 (44)	10.3 (52)	0.403
All the previous [correct]	17.1 (86)	20.0 (101)	0.199
Doesn’t know	42.3 (213)	30.2 (152)	<0.001
HCV treatment knowledge sub-score, %^a^			
mean ± SD	62.4 ± 26.9 %	76.8 ± 18.3 %	<0.001
median (min-max)	69.4 (0.0–100)	80.6 (0.0–100)	
*Questions*			
10. What are the treatment options for HCV disease, % (n)			<0.001
Treatment that should be used over the lifetime	3.6 (18)	1.8 (9)	
Treatment that should be used during a limited period [correct]	82.7 (417)	92.7 (467)	
There is no treatment	1.8 (9)	0.4 (2)	
Doesn’t know	11.9 (60)	5.2 (26)	
11. What are the possible treatment outcomes, % (n)			<0.001
Most patients develop liver cirrhosis	5.0 (25)	2.2 (11)	
Most patients become cured [correct]	66.7 (335)	86.1 (434)	
Doesn’t know	28.3 (142)	11.7 (59)	
12. What are main adverse reactions to HCV treatment, % (n)^d^			
Flu-like symptoms [correct]	35.3 (178)	45.8 (231)	<0.001
Fatigue [correct]	53.0 (267)	65.3 (329)	<0.001
Depression [correct]	36.7 (185)	50.8 (256)	<0.001
Appetite loss [correct]	42.3 (213)	54.0 (272)	<0.001
Nausea, vomits and diarrhea [correct]	33.7 (170)	44.8 (226)	<0.001
Skin reactions [correct]	15.5 (78)	27.2 (137)	<0.001
Weight loss [correct]	40.7 (205)	60.3 (304)	<0.001
Alopecia [correct]	21.4 (108)	32.1 (162)	<0.001
Doesn’t know	31.0 (156)	6.2 (31)	<0.001
Other	6.0 (30)	16.1 (81)	
13. Do adverse reactions disappear after treatment discontinuation			
Yes, % (n) [correct]	68.8 (346)	85.5 (431)	<0.001

McNemar test was used to compare changes in the proportion of correct answers for each question between baseline and after program implementation. Wilcoxon test was used to analyze changes in overall knowledge score and sub-scores between both time points. *p* < 0.05 is considered statistically significant

*HEP* Health Educational Program, *SD* standard-deviation

^a^The overall knowledge score was calculated by assigning one point to each correct answer. The score reflected the percentage of points obtained out of the 13 questions. For all questions, missing data and “don’t know” answers were considered as not correct (score = 0). Disease knowledge sub-score was calculated based on correct answers to questions 1–9. HCV treatment knowledge sub-score was calculated based on correct answers to questions 10–13

^b^Whenever a patient chose more options than the correct one, a score of “1/number of answers” was attributed

^c^Whenever a patient chose one or two out of three correct options a score of 0.33 and 0.67 was attributed, respectively

^d^The score was the number of correct answers over nine questions

Overall knowledge score increased from 69 % to 79 % (*p* < 0.001). A statistically significant improvement was observed in the disease knowledge sub-score (from 72 % to 80 %; *p* < 0.001). The awareness that symptoms do not appear immediately after infection with HCV increased from 78 % at baseline to 89 % after the HEP (*p* < 0.001). Knowledge also improved regarding the higher chance of progression to cirrhosis if disease is left untreated (77 % to 86 %, *p* < 0.001), of increased progression in the case of HIV co-infection (from 79 % to 87 %, p < 0.001) and of alcohol consumption (97 % to 99 %, *p* = 0.004). Improvements were also observed regarding HCV physiopathology (from 92 % to 96 %, *p* < 0.001), transmission mode (knowledge that HCV is both sexually and blood transmitted increased from 74 % to 84 %, *p* < 0.001) and the possibility of cure (from 74 % to 89 %, *p* < 0.001). Comparing to baseline, more patients have identified at least one HCV symptom (*p* < 0.001) and at least one disease most associated with HCV (*p* < 0.001). The knowledge that HIV is commonly associated with HCV has also improved significantly (from 20 % to 32 %, *p* < 0.001).

There was a statistically significant improvement in the HCV treatment knowledge sub-score (from 62 % to 77 %; *p* < 0.001). There was an increase in the proportion of patients knowing that treatment is used during a limited period (from 83 % to 93 %, *p* < 0.001) and that most patients can be cured (from 67 % to 86 %, *p* < 0.001). Knowledge about the main side effects of HCV treatment and their resolution after discontinuation also improved (from 69 % to 86 %, *p* < 0.001).

### Factors associated with baseline knowledge

Overall knowledge score at baseline was higher among female participants (mean score: 70.9 % vs. 68.2 % in males; *p* = 0.050) and among patients with higher educational level (<5 years: 59.8 %; 5–9 years: 67.8 %, ≥10 years: 76.5 %; *p* < 0.001). Knowledge was also higher among patients who were using injectable drugs (78.2 % vs. 68.4 %; *p* = 0.008), who had been referred for treatment (70.8 % vs. 64.4 %, *p* < 0.001), who had received prior HCV treatment (75.0 % vs. 67.1 %; *p* < 0.001), and who were currently referred to a liver specialist (70.5 % vs. 66.4 %, *p* = 0.006).

The linear regression model (Table 5) showed that a higher baseline knowledge score was independently associated with educational level ≥ 10 years (regression coefficient [B] = 15.13, 95 % CI [10.42–19.84]), current use of intravenous drugs (B = 7.99, 95 % CI [0.44–15.56]), previous referral for treatment (B = 4.27, 95 % CI [1.10–7.43]) and prior HCV treatment (B = 5.40, 95 % CI [1.82–8.97]).

**Table 5:** Multiple linear regression to investigate the association between patient characteristics and overall knowledge at baseline

	Overall knowledge score at baseline, %	Optimized model^a^		
	mean ± SD^b^	B	95 % CI for B	*p* value
Educational level				
<5 years	59.8 ± 19.3	Reference		
5 – 9 years	67.8 ± 16.1	7.11	[2.96–11.26]	0.001
≥10 years	76.5 ± 12.8	15.13	[10.42–19.84]	<0.001
Current use of intravenous drugs				
Yes	78.2 ± 13.1	7.99	[0.44–15.56]	0.038
No	68.4 ± 16.6	Reference		
Previous referral for treatment				
Yes	70.8 ± 16.3	4.27	[1.10–7.43]	0.008
No	64.4 ± 16.7	Reference		
Previous HCV treatment				
Yes	75.0 ± 15.0	5.40	[1.82–8.97]	0.003
No	67.1 ± 16.7	Reference		

*p* < 0.05 is considered statistically significant. Variables not introduced in the final model did not show statistical significance

*B* Regression coefficient, *95 % CI for B* 95 % confidence interval for the regression coefficients

^a^The variables sex, educational level, current use of intravenous drugs, previous referral for treatment, previous HCV treatment, and current referral to liver specialist and number of hospital visits in the previous 6 months were included in the initial model

^b^Unadjusted mean and standard-deviation (SD) of overall knowledge score at baseline

### Clinical care of HCV

A statistically significant increase in the patient referral rate between the baseline and post-educational assessment was observed (from 40.2 % to 49.6 %; *p* < 0.001), while the treatment proposal rate dropped from 60.1 % to 52.0 % (*p* < 0.001) (Table 6). The increase found in the treatment initiation rate (from 47.5 % to 57.7 %) was not statistically significant. There were no changes in the treatment retention rate (75.9 % vs. 76.0 %).

**Table 6:** HCV infection clinical management prior and after exposure to HEP

Endpoint	Baseline % (n/N)	Post-HEP % (n/N)	*p* value
Patient referral rate	56.2 % (283/504)	67.5 % (340/504)	<0.001
95 % CI	51.8–60.5 %	63.4–71.6 %	
Attendance at referral rate	74.4 % (203/273)	73.5 % (250/340)	0.904
95 % CI	69.2–79.5 %	68.8–78.2 %	
Treatment proposal rate	60.1 % (122/203)	52.0 % (130/250)	<0.001
95 % CI	53.4–66.8 %	45.8–58.2 %	
Treatment initiation rate	47.5 % (58/122)	57.7 % (75/130)	0.114
95 % CI	38.7–56.4 %	49.2–66.2 %	
Treatment retention rate	75.9 % (44/58)	76.0 % (57/75)	0.108
95 % CI	64.8–86.9 %	65.2–84.3 %	

All comparisons were made with McNemar test. *p* < 0.05 is considered statistically significant

95 % CI = 95 % confidence interval

*n* number of patients that achieved the endpoint, *N* number of patients from which the rates (%) were calculated

*HEP* Health Educational Program

## Discussion

This study aimed primarily to investigate the impact of a multidimensional educational program implemented at Portuguese SDTCs, on HCV patients’ knowledge about the disease and its treatment, and to explore the factors most associated with this outcome.

Overall, there was a statistically significant increase in patient knowledge after exposure to the HEP. This trend was also observed for the specific domains of disease and HCV treatment (sub-scores). Our findings are similar to those observed in other educational programs. For instance, Surjadi *et al.* [12] observed that HCV patient’s mean percent knowledge improved significantly after a two-hour educational session. Norton *et al*. [30] have observed that, though baseline HCV knowledge was poor in HCV high-risk individuals, a brief on-site educational intervention improved both knowledge and acceptability of HCV care. More recently, Zemersky *et al.* [31] reported significant improvements in patients' knowledge about hepatitis C after a two-session educational intervention.

In addition, we found that higher educational level (≥10 years), current use of intravenous drugs, previous exposure to HCV treatments and current referral to treatment were associated with higher overall knowledge at baseline. Strauss *et al.* [32] reported that PWID scored higher on a HCV knowledge assessment than non-drug users. Other studies have observed that knowing someone with HCV and seeing a physician after their first positive HCV test were factors associated with higher knowledge [30, 33]. Our findings are aligned with others results and suggest that, besides a good educational background, the accumulated experience with HCV disease may contribute to patient increased awareness about this condition and its treatment.

Regarding HCV clinical care outcomes, we observed a statistically significant increase in the rate of referral to the liver specialist after the implementation of HEP. The baseline referral rate observed in our study (56.2 %) is similar to that reported in the literature, varying from 30 % to 50 % [20, 34, 35]. After exposure to the HEP, the referral rate reached approximately 68 %. Nonetheless, this was not accompanied by a statistically significant increase in the treatment initiation rate. In fact, the rate of treatment proposal decreased markedly. We cannot exclude that this decrease may be due to the fact that some of the patients assessed during phase I may have initiated treatment during phase II. Still, the difference in treatment initiation rates between evaluations was not statistically significant and, since there was an increase in referral rate at phase II, one may expect treatment proposal rate to increase or maintain in the absence of other treatment constraints. These results are aligned with the work of Brugmann *et al*. [7], who reported a decline in the number of patients treated for HCV in Portugal between 2011 and 2013 [7, 36]. Studies suggest that treatment costs and frequent clinical and psychological comorbidities can be important barriers to HCV treatment access [37–40]. This finding may also be attributable to the persisting resistance of physicians in treating a patient population presumably unstable and difficult to treat. In fact, many liver specialists remain hesitant to prescribe a costly and potentially intolerable therapy to active drug users, who are still considered at higher risk of non-adherence and reinfection [8, 41].

We found that treatment retention remained stable. However, one should be cautious in interpreting these results due to the relatively short-term follow up of the participants and uncertain duration of patient knowledge. Research shows that HCV patients often require additional support from healthcare providers to initiate and maintain their treatment, and that longer studies provide a better estimate of adherence outcomes [42, 43].

We cannot exclude the presence of selection bias, as PWID recently referred for treatment are often more involved with the treatment community members, regularly visiting SDTCs and, therefore, are more exposed to educational initiatives [32]. Nonetheless, we found room for improvement in the knowledge, namely regarding the most common symptoms of the disease and treatment side effects. Future educational initiatives should specially focus on these aspects.

Since we did not collect the number of participants that attended each workshop, it was not possible to characterize the patient adherence to workshops. Nevertheless, all patients were exposed to the educational videos and leaflets provided at the STDCs. It is not likely that the literacy level of the participants may have affected their understanding of the questions as these were orally presented by the investigator who clarified eventual doubts regarding terminology. To minimize differences between participating centers, investigators from each STDCs received training related with the administration of questionnaires and workshops. Regarding retention bias, patients who did not complete phase II evaluation were approximately 2 years younger than patients who completed both evaluations and showed a lower unemployment rate. It may be that professionally active patients were less likely to attend STDC during phase II. Finally, study data were collected before the emergence of direct-acting antiviral agents and, therefore, it is likely that other treatment barriers could emerge associated to these regimens [17, 44].

Few studies have looked at the impact of an educational intervention among PWID with HCV. The strengths of this study are the relatively large sample and of the involvement of several centers, enhancing the generalizability of the findings. The fact that healthcare professionals were engaged in several educational strategies, including the discussion of clinical cases between professionals from STDCs and liver specialists, was another strength of the HEP as it represented an opportunity to strengthen communication between healthcare providers and, thus, patient referral to HCV treatment [7].

Evidence shows that HCV-infected individuals have limited knowledge about the disease and that improving this knowledge can influence a patient’s decision to initiate treatment [10, 45]. Furthermore, knowledge increases can lead to prevention of infection and social integration in this setting. A review concluded that HCV infected patients benefited from educational interventions, with six out of the ten selected studies reporting significant improvements in patient-related outcomes such as disease knowledge, behavioral changes and willingness to start and complete treatment [12, 46].

Though treatment options and success rates have evolved significantly in recent years, effective management of hepatitis C among drug users, educational strategies are still essential to improve patient knowledge about the disease and thus optimize treatment outcomes [47]. SDTCs represent an ideal setting to engage with patients and to implement health education programs with drug users and PWID, which are frequently considered as hard-to-reach populations [37, 48].

## Conclusions

Our study showed a multidimensional HEP conducted at STDCs improved significantly patient knowledge about hepatitis C. In fact, this educational program has contributed to increase disease- and treatment-related knowledge, even though overall baseline knowledge was already high and associated with educational level, current use of intravenous drugs, previous exposure to HCV treatments and current reference to treatment. In addition, the educational program increased the rate of referral to the liver specialist and showed a great potential to support healthcare professionals in managing HCV. During the era of direct-acting antiviral agents, further educational initiatives should focus on populations with poor educational background and less experienced with the disease, while evaluating barriers to treatment initiation.

## Additional files

Additional file 1: This file contains the English language version of the questionnaire used in the study. (PDF 51 kb)
Additional file 2: This file contains the raw data for the variables presented in this manuscript. (XLSX 238 kb)

## Abbreviations

CHC: Chronic hepatitis C
CI: Confidence interval
HCV: Hepatitis C virus
HEP: Health educational program
OR: Odds ratio
PWID: People who inject drugs
SD: Standard-deviation
SDTCs: Substance dependence treatment centers
