# Supplementary material for: A multidimensional education program at substance dependence treatment centers improves patient knowledge and hepatitis C care
Source: BMC Infect Dis. 2016 Oct 12;16:565. doi: 10.1186/s12879-016-1883-6 (PMC5062838; doi:10.1186/s12879-016-1883-6)
Supplement: Additional file 1: — This file contains the English language version of the questionnaire used in the study. (PDF 51 kb) [file 12879_2016_1883_MOESM1_ESM.pdf]

STUDY QUESTIONNAIRE

ELIGIBILITY AND DEMOGRAPHICS	
1.1. Visit date<br>1.2. Informed consent date	|__|__|__|__|-|__|__|-|__|__| (yyyy-mm-dd)<br>|__|__|__|__|-|__|__|-|__|__| (yyyy-mm-dd)
2. Eligibility – inclusion criteria<br>2.1. Age ≥ 18 years<br>2.2. Patient with HCV infection with an appointment at the STDC during the evaluation period<br>2.3. Patient provide Informed Consent	 [ ] Yes [ ] No<br>[ ] Yes [ ] No<br>[ ] Yes [ ] No
3. Eligibility – inclusion criteria<br>3.1. Patients without confirmed diagnosis of HCV infection, registered in the STDC	 [ ] Yes [ ] No
4. Demographic Data<br>4.1. Date of Birth (year and month)<br>4.2. Sex	 |__|__|__|__|-|__|__| (yyyy-mm)<br>[ ] Male [ ] Female
4.3. Educational level	[ ] No educational level<br>[ ] 1st year [ ] 10th year<br>[ ] 2nd year [ ] 11th year<br>[ ] 3rd year [ ] 12th year<br>[ ] 4th year [ ] BSc<br>[ ] 5th year [ ] Degree<br>[ ] 6th year [ ] Master<br>[ ] 7th year [ ] Phd<br>[ ] 8th year<br>[ ] 9th year
4.4. Marital status	[ ] Single<br>[ ] Married<br>[ ] Divorced<br>[ ] Widow
4.5. Professional status	[ ] Student<br>[ ] Unemployed<br>[ ] Employed, profession: ___________________________<br>[ ] Retired, profession: ___________________________<br>[ ] Other, specify: _______________________________
CLINICAL CHARACTERISTICS	
1. Current status of substance abuse	[ ] Intravenous drug use<br>[ ] Non-intravenous drug use<br>[ ] Alcohol abuse<br>[ ] Opioid substitution treatment<br>[ ] Other, specify: _______________________________<br>[ ] Unknown

2. Date of diagnosis of HCV infection (year and month)	|__|__|__|__|-|__|__| (yyyy-mm)<br>[ ] Doesn't know
3. HCV genotype	[ ] 1<br>[ ] 1a<br>[ ] 1b<br>[ ] 2<br>[ ] 3<br>[ ] 4<br>[ ] Unknown
4. HCV viral load (x10^6 IU/ml)	______________<br>[ ] Unknown
5. HCV Transmission Mode	[ ] Intravenous drug use<br>[ ] Post-transfusion<br>[ ] Peri-natal<br>[ ] Sexual<br>[ ] Other, specify: ____________________________<br>[ ] Doesn't know
6. Comorbities<br>6.1. HIV<br>6.2. HBV<br>6.3. Mental disorders<br>6.3.1. (if yes) Specify (considering ICD-10)<br>6.4. Other comorbidities<br>6.4.1. (if yes) Specify	 [ ] Yes [ ] No [ ] Doesn't know<br>[ ] Yes [ ] No [ ] Doesn't know<br>[ ] Yes [ ] No [ ] Doesn't know<br>____<br>[ ] Yes [ ] No [ ] Doesn't know<br>____
HEPATITIS C TREATMENT	
1. Patient was previous referred to treatment?<br>2. Patient received previous treatment?	[ ] Yes [ ] No [ ] Doesn't know<br>[ ] Yes [ ] No [ ] Doesn't know
3. Patient is currently referred to a hospital clinic?<br>3.1. (if yes) Which hospital?<br>3.1.1. This current referral has resulted in a medical appointment with a liver specialist at that hospital?<br>3.2. (if no) Patient was referred to a hospital clinic during this evaluation?<br>3.2.1 (if yes) Which hospital?	[ ] Yes [ ] No [ ] Doesn't know<br>___________________________<br>[ ] Yes [ ] No [ ] Doesn't know<br>[ ] Yes [ ] No [ ] Doesn't know<br>___________________________
4. Did the liver specialist (at the hospital) prescribed the patient with HCV treatment?<br>4.1. (if yes) What is the current treatment status?	[ ] Yes [ ] No [ ] Doesn't know<br>[ ] Patient has not initiated treatment<br>[ ] Initiated and is still under treatment<br>[ ] Initiated and stopped treatment due to unknown reason<br>[ ] Initiated and stopped treatment due to lack of effectiveness<br>[ ] Initiated and stopped treatment due to adverse events<br>[ ] Initiated and stopped treatment due to lack of persistence<br>[ ] Initiated and completed treatment as prescribed<br>[ ] Doesn't know
5. How many medical appointments at the hospital did the patient attended, in the last 6 months?	___________________________
6. Number of visits at the STDC in the last 6 months?	___________________________

PATIENT KNOWLEDGE ABOUT HEPATITIS C	
1. How does the Hepatitis C infection evolves?	
1.1. Patient shows symptoms immediately after infection with HCV?	[ ] Yes [ ] No [ ] Doesn't know
1.2. If left untreated, the majority of cases progress to cirrhosis?	[ ] Yes [ ] No [ ] Doesn't know
1.3. Co-infection with HIV may increase disease progression?	[ ] Yes [ ] No [ ] Doesn't know
1.4. Alcohol consumption may increase disease progression?	[ ] Yes [ ] No [ ] Doesn't know
2. What is Hepatitis C disease?	[ ] Is a liver viral disease<br>[ ] Is a disease that affects whole body organs<br>[ ] Doesn't know
3. How is Hepatitis C transmitted?	[ ] Sexual transmission only<br>[ ] Sexual and blood transmission<br>[ ] Physical contact<br>[ ] Sharing of materials related with drug use<br>[ ] Doesn't know<br>[ ] Other, specify: ______________________________
4. What are the symptoms of Hepatitis C?	[ ] Generalized weakness<br>[ ] Weight loss<br>[ ] Vomits and diarrhea<br>[ ] All the previous<br>[ ] There are no symptoms<br>[ ] Doesn't know
5. Hepatitis C is curable?	[ ] Yes [ ] No [ ] Doesn't know
6. Which diseases are most associated with Hepatitis C?	[ ] HIV<br>[ ] Hepatitis B<br>[ ] Tuberculosis<br>[ ] All the previous<br>[ ] Doesn't know
7. What are the treatment options for Hepatitis C?	[ ] Treatment that should be used over the lifetime<br>[ ] Treatment that should be used during a limited period<br>[ ] There is no treatment<br>[ ] Doesn't know
8. What are the possible treatment outcomes?	[ ] Most patients develop liver cirrhosis<br>[ ] Most patients become cured<br>[ ] Doesn't know
9. What are main adverse reactions to Hepatitis C treatment?	[ ] Flu-like symptoms<br>[ ] Fatigue<br>[ ] Depression<br>[ ] Appetite loss<br>[ ] Nausea, vomits and diarrhea<br>[ ] Skin reactions<br>[ ] Weight loss<br>[ ] Alopecia<br>[ ] Doesn't know<br>[ ] Other, specify: ______________________________
10. Do adverse reactions disappear after treatment discontinuation?	[ ] Yes [ ] No [ ] Doesn't know
